# Three new species of *Lachemilla* (Rosaceae) from South America

**DOI:** 10.3897/phytokeys.127.36324

**Published:** 2019-07-19

**Authors:** Diego F. Morales-Briones, Katya Romoleroux, David C. Tank

**Affiliations:** 1 Department of Plant and Microbial Biology, University of Minnesota, 140 Gortner Laboratory, 1479 Gortner Avenue, Saint Paul, Minnesota, 55108, USA University of Idaho Moscow United States of America; 2 Department of Biological Sciences and Stillinger Herbarium, University of Idaho, 875 Perimeter Drive MS 3051, Moscow, Idaho 83844-3051, USA University of Minnesota Saint Paul United States of America; 3 Herbario QCA, Escuela de Ciencias Biológicas, Pontificia Universidad Católica del Ecuador, Av. 12 de Octubre 1076 y Roca, Apartado 17-01-2184, Quito, Ecuador Pontificia Universidad Católica del Ecuador Roca Quito Ecuador

**Keywords:** Allopolyploidy, Colombia, *
Lachemilla
*, new species, páramo, Peru, Rosaceae

## Abstract

Three new species of *Lachemilla* (Rosaceae), two from Colombia and one from Peru, are described and illustrated. *Lachemillarothmaleriana* is characterized by its stout stems, sericeous-villous indumentum, and wide ascending sheaths with trilobate lateral lobes. *Lachemillaargentea* presents a unique combination of tripartite basal leaves with an adaxial silvery villous indumentum, and decumbent branches with verticillate lobed sheaths. Finally, *Lachemillacyanea* has distinctly basal reniform leaves with a blue-green color and hirsute pubescence. Phylogenetic analyses of the nuclear ribosomal cistron and multiple regions of the plastid genome revealed the allopolyploid origin of the three new taxa.

## Abstract (es)

Tres nuevas especies de *Lachemilla* (Rosaceae), dos de Colombia y una de Perú, se describen e ilustran. *Lachemillarothmaleriana* se caracteriza por tener tallos robustos, indumento sericeo-villoso, y verticilos anchos ascendentes con lóbulos laterales trilobados. *Lachemillaargentea* presenta una combinación única de hojas basales tripartitas con indumento villoso plateado en el haz y ramas decumbentes con verticilos lobulados. Finalmente, *Lachemillacyanea* presenta hojas basales distintivas de forma reniforme, color azul–verdoso y pubescencia hirsuta. Análisis filogenéticos del cistrón ribosomal nuclear y múltiples regiones del genoma cloroplástico revelaron el origen alopoliploide de los tres nuevos taxones.

## Introduction

The genus *Lachemilla* (Focke) Rydb. is a morphologically diverse group that includes perennial herbs, subshrubs, and dwarf shrubs (Romoleroux 1996, 2004; Gaviria 1997). *Lachemilla* is distributed in the neotropical mountains from Mexico to Argentina and Chile, between 2,200 m and 5,000 m (Romoleroux 1996, 2004; Gaviria 1997), and it is especially common and diverse in the high elevation ecosystems of the Northern Andes where this group has undergone a rapid ecological radiation associated with the most recent Andean orogeny (Romoleroux 2004; Morales-Briones et al. 2018a).

The taxonomy of *Lachemilla* has proven challenging (e.g., Perry 1929; Rothmaler 1935a, 1935b, 1937), and although more recent regional treatments have reviewed this group (Gaviria 1997; Romoleroux 2004; Barrie 2015), the complex morphology of *Lachemilla* has obscured species boundaries and morphological subdivisions. Additionally, recent attention to the systematics of *Lachemilla* has resulted in the description of several new species (Romoleroux 2009; Romoleroux and Morales-Briones 2012; Morales-Briones 2016), but a comprehensive monographic revision of the genus is still needed (Romoleroux and Morales-Briones in prep.).

Based on molecular phylogenetic evidence, Morales-Briones et al. (2018a) proposed the recognition of 61 species of *Lachemilla* separated into four well-supported lineages that are somewhat congruent with previously morphology-based classification systems (Perry 1929; Rothmaler 1937). The Tripartite clade includes herbs with ascending and procumbent stems and tripartite leaves. The Verticillate clade includes subshrubs with erect or decumbent stems and reduced leaves that fuse with the stipules to form verticillate sheaths. The Pinnate clade comprises species with repent or decumbent stems and pinnate or bipinnatifid basal leaves. Finally, the Orbiculate clade encompasses species with a stoloniferous habit and palmately lobed leaves, and, it has been established that the Orbiculate clade is of ancient hybrid origin (Morales-Briones et al. 2018b). Moreover, Morales-Briones and Tank (2019a), using copies (or ribotypes) of the nuclear ribosomal (nrDNA) cistron and multiple regions of the plastid genome (cpDNA), showed that at least 30 species of *Lachemilla* are allopolyploids, and that this condition is widespread among the four main clades of this group.

Here, we describe and illustrate three new species of *Lachemilla*, two from Colombia and one from Peru, and using a phylogenetic approach, we show evidence that these new species are of allopolyploid origin.

## Materials and methods

### Phylogenetic analyses

We used previously published datasets from Morales-Briones and Tank (2019b) from the nrDNA cistron and 45 regions of cpDNA for *Lachemilla*, and included 68 samples representing 48 species of *Lachemilla*, seven samples of the three new taxa, and four outgroups (Appendix 1). DNA extraction, amplification and sequencing were carried out as described in Morales-Briones and Tank (2019a). Molecular datasets from Morales-Briones and Tank (2019a) and data for the new taxa presented here were generated simultaneously. Data processing and ribotype selection was performed as described in Morales-Briones and Tank (2019a). The three regions of the nrDNA (ETS, ITS1, and ITS2) were concatenated, and the 45 regions of the cpDNA were concatenated as well. Phylogenetic analyses of the nrDNA and cpDNA matrices were carried out independently to avoid conflicting phylogenetic signal due to widespread cytonuclear discordance in *Lachemilla* produced by polyploidy and ancient hybridization (Morales-Briones et al. 2018a, 2018b). Maximum likelihood (ML) analyses were conducted with RAxML v8.2.10 (Stamatakis 2014) using a GTRGAMMA model, 100 searches for the best tree, and clade support was assessed with 1,000 bootstrap replicates summarized using transfer bootstrap expectation (TBE; Lemoine et al. 2018). Bayesian inferences (BI) were performed with MrBayes v3.2.6 (Ronquist et al. 2012) on the CIPRES portal (Miller et al. 2010). Analyses consisted of two independent runs with four Markov Chain Monte Carlo (MCMC) chains for 20 million generations with trees sampled every 20,000^th^ generation and allowing sampling across the entire substitution rate model space using reversible-jump Markov Chain Monte Carlo (rjMCMC) (nst = mixed) and rate variation set to GAMMA. Parameter estimate convergence of the independent MCMC runs was assessed by analyzing plots of all parameters and the –lnL after reaching an ESS (effective-sample size) ≥ 200 using Tracer v1.6 (Rambaut et al. 2014). A 50% majority rule consensus tree was generated and posterior probabilities (PP) were calculated after removing the first 25% of sampled trees. Alignments and phylogenetic trees are available at TreeBASE (http://purl.org/phylo/treebase/phylows/study/TB2:S24437).

### Taxonomic analyses

Morphological characters were studied using dried herbarium specimens of *Lachemilla* deposited in ANDES, CAS, COL, F, HUT, HAO, ID, JE, MEXU, MO, NY, QCA, TEX, and USM. Additionally, we reviewed species descriptions and types to determine the existence of the new taxa. The conservation status of the new species was evaluated using the guidelines of the International Union for Conservation of Nature (IUCN 2017).

## Results and discussion

### Phylogenetic analyses

**Figure 1.**
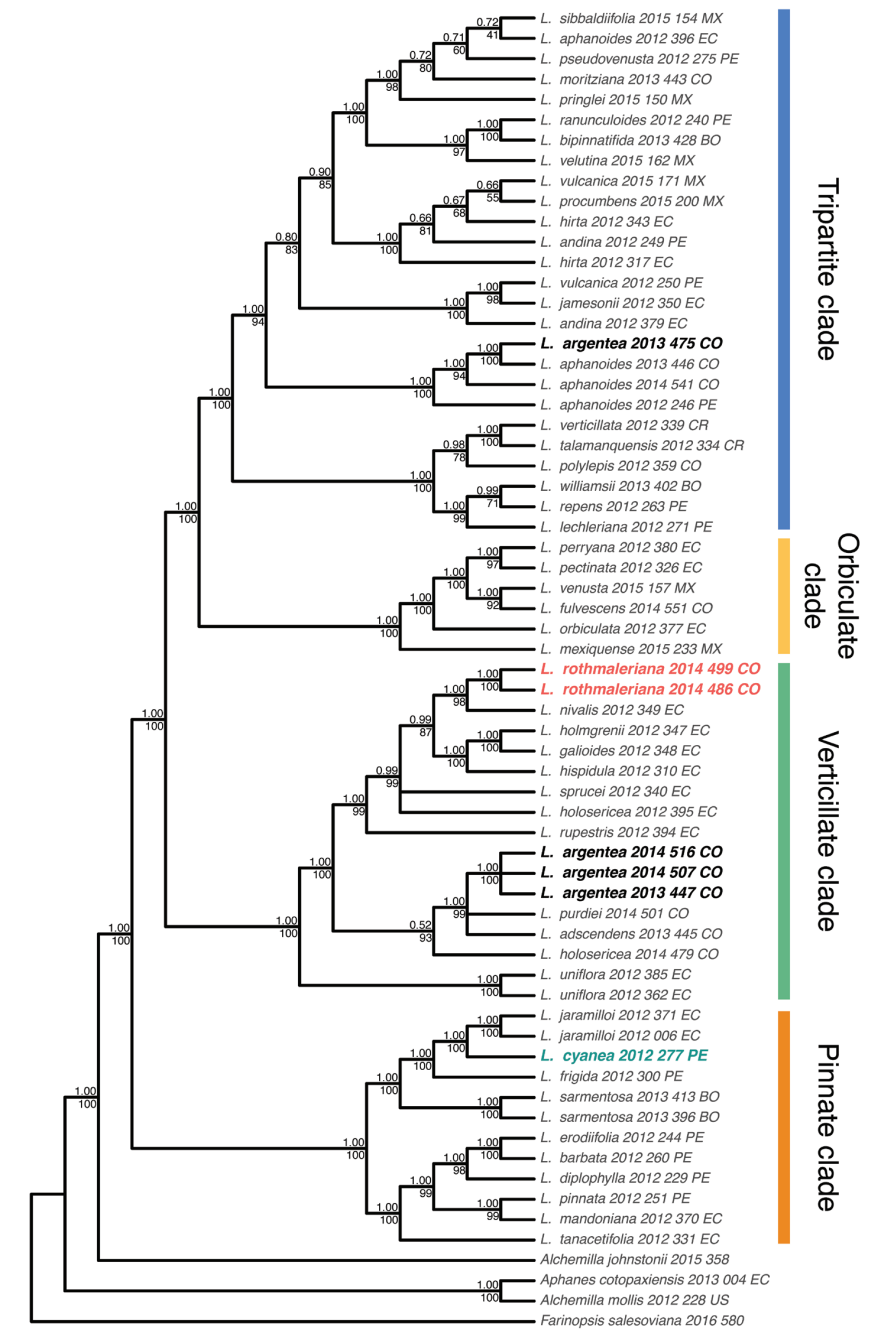
Bayesian 50% majority rule consensus tree of the cpDNA dataset. Posterior probabilities and transfer bootstrap expectation support values are shown above and below the branches, respectively.

The final cpDNA concatenated matrix included 65 sequences representing 47 species of *Lachemilla* and four outgroup species, and had an aligned length of 22,000 bp. The ML and BI analyses recovered the same overall topology (Fig. 1). The four major clades within *Lachemilla* are the same as in previous phylogenetic analyses of the genus (Morales-Briones et al. 2018a; Morales-Briones and Tank 2019a). The samples of *L.rothmaleriana* form a clade (TBE = 100, PP = 1.0) and are placed as sister to *L.nivalis* (Kunth) Rothm. in the Verticillate clade. *Lachemillacyanea* is placed in the clade (TBE = 100, PP = 1.0) composed of *L.frigida* (Wedd.) Rothm. and *L.jaramilloi* Romol. & D.F. Morales-B. (a known allopolyploid species) within the Pinnate clade. *Lachemillaargentea* is placed in two major clades. Three samples form a clade (TBE = 100, PP = 1.0) that is placed along other allopolyploid species (*L.purdiei* (L.M. Perry) Rothm., *L.adscendens* (Rothm.) Rothm. and *L.holosericea* (L.M. Perry) Rothm.) within the Verticillate clade. The remaining sample (2013_475_CO) is placed within a clade composed of only samples of *L.aphanoides* (Mutis ex L. f.) Rothm. within the Tripartite clade.

**Figure 2.**
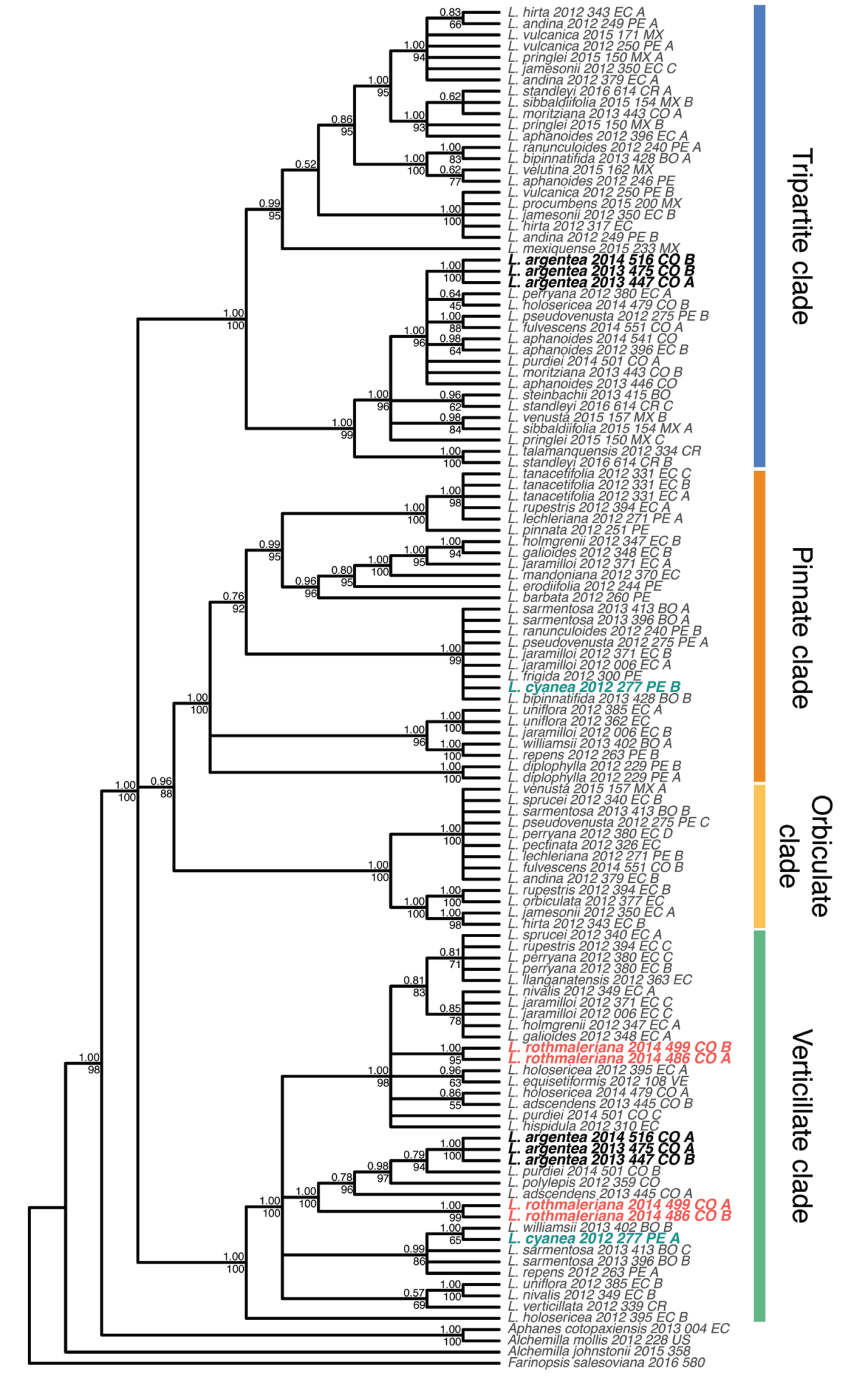
Bayesian 50% majority rule consensus tree of the nrDNA dataset. Posterior probabilities and transfer bootstrap expectation support values are shown above and below the branches, respectively.

The final nrDNA concatenated alignment included 121 sequences representing 51 species of *Lachemilla* and four outgroup species, and had aligned length of 1038 bp. The ML and BI analyses also recovered the same overall topology (Fig. 2). As in the cpDNA analyses, the four major clades within *Lachemilla* are the same as in previous studies (Morales-Briones et al. 2018a; Morales-Briones and Tank 2019a). *Lachemillarothmaleriana* showed the presence of two ribotypes within the Verticillate clade. One of the ribotypes is placed as sister (TBE = 100, PP = 1.00) to a clade composed of *L.adscendens*, *L.polylepis* (Wedd.) Rothm., *L.purdiei*, and *L.argentea*. The second copy is placed within a clade (TBE = 98, PP = 0.91) that includes several species with verticillate sheaths along the entire stem, like *L.hispidula* (L.M. Perry) Rothm., *L.equisetiformis* (Trevir.) Rothm., and *L.nivalis*, and several allopolyploid species (e.g. *L.adscendens*, *L.holosericea*, *L.sprucei* (L.M. Perry) Rothm.), but the resolution within this clade is rather uncertain. *Lachemillacyanea* also presents two ribotype copies. One copy, as in the cpDNA tree, is placed in the clade (TBE = 100, PP = 0.99) composed of *L.frigida* and its allopolyploid species (e.g. *L.jaramilloi*, *L.sarmentosa* (L.M. Perry) Rothm.) within the Pinnate clade. The other ribotype is placed within a clade (TBE = 86, PP = 0.99) composed only of allopolyploid species (*L.williamsii* (L.M. Perry) Rothm., *L.repens* (C. Presl) Rothm., and *L.sarmentosa*) within the Verticillate clade. All samples of *L.argentea* also have two ribotypes. The first is placed in the Tripartite clade along several allopolyploid species that involve *L.aphanoides* as one parental species (e.g. *L.pseudovenusta*, *L.fulvescens* (L.M. Perry) Rothm., *L.purdiei*, *L.perryana* (Rothm.) Rothm.). The second copy is placed as sister (TBE = 84, PP = 0.79) to the allopolyploid species *L.purdiei* in the Verticillate clade.

Previous phylogenetic analysis of *Lachemilla* have shown that allopolyploidy is common in this group with at least 30 species confirmed to be of allopolyploid origin (Morales-Briones and Tank 2019a). Here we show that the three new taxa of *Lachemilla* described here are also allopolyploids. *Lachemillarothmaleriana* appears to be an allopolyploid of two different species within the Verticillate clade. Based on the cpDNA tree we can determine that *L.nivalis* might be the maternal lineage, while the paternal lineage seems to be the same as in the allopolyploids *L.adscendens*, *L.purdiei* and *L.argentea*, all from Colombia. From the nrDNA tree we can see that the paternal species is either extinct or unsampled. Morphologically, *L.rothmaleriana* presents the main characteristic of the Verticillate clade, which is the presence of reduced leaves that fuse with the stipules to form verticillate sheaths, but this new taxon is distinguished mainly by the presence of wide ascending sheaths with trilobate lateral lobes and a dense sericeous-villous indumentum (see Taxonomic treatment for details). The presence of divided lobes is a characteristic of other species of the Verticillate clade that are now known to be of hybrid origin, like *L.adscendens* and *L.sprucei* (Morales-Briones and Tank 2019a).

*Lachemillaargentea* is also an allopolyploid species of two major clades. The cpDNA tree shows that three of the four samples have the maternal lineage from the Verticillate clade, while for one sample (2013_475_CO) the maternal lineage is *L.aphanoides* in the Tripartite clade. The nrDNA data shows that the paternal lineage of *L.argentea* is in the Tripartite clade, except for 2013_475_CO which has an extinct or unsampled parental lineage in the Verticillate clade. Although the sample 2013_475_CO is monophyletic with the other samples of this species for both ribotypes in the nrDNA, the different position in the cpDNA tree is clear evidence that the parental contribution in the formation of allopolyploid species in *Lachemilla* can work in both directions, as well as the recurrent origin of allopolyploids, which has also been previously shown for this group (Morales-Briones and Tank 2019a), and is known for other related groups in Rosaceae, like *Fragaria* (Dillenberger et al. 2018). The basal tripartite leaves of *Lachemillaargentea* are characteristic of the Tripartite clade, but its decumbent branches with verticillate lobed sheaths resemble the Verticillate clade (see Taxonomic treatment for details).

Finally, *L.cyanea* also shows evidence of an allopolyploid origin between two major clades. The maternal species appears to be *L.frigida* in the Pinnate clade, which is also the maternal lineage of *L.jaramilloi* (Morales-Briones and Tank 2019a), with which *L.cyanea* also shares a rosette habit (see Taxonomic treatment for details). The paternal lineage appears to be the same as in the allopolyploid and morphologically similar species *L.williamsii*, *L.repens*, and *L.sarmentosa* in the Verticillate clade, where the parental lineage of these species has been shown likely to be extinct (Morales-Briones and Tank 2019a). Interestingly, these three similar species are hybrids of three different major clades (Pinnate, Verticillate, and Tripartite; Morales-Briones et al. 2018a). Given its tripartite leaf and glomerulate inflorescence (see Taxonomic treatment for details), *L.cyanea* also shares characteristics with the Tripartite clade, but we were not able to obtain evidence of this from the nrDNA tree, which can be explained by omission nrDNA copies during bioinformatic processing of the PCR amplicon pools as seen in other species of *Lachemilla* (Morales-Briones and Tank 2019a).

### Taxonomic treatment

#### Lachemilla rothmaleriana

Taxon classificationPlantaeRosalesLachemilla

D.F.Morales-B. & Romol. sp. nov.

urn:lsid:ipni.org:names:77199640-1

Figs 3 , 4 , 5

##### Diagnosis.

*Lachemillarothmaleriana* differs from *L.hispidula* (L.M. Perry) Rothm. and *L.nivalis* (Kunth) Rothm. by its stout stems, sericeous-villous indumentum, wide ascending sheaths with trilobate lateral lobes, and a turbinate-campanulate hypanthium.

##### Type.

**COLOMBIA. Boyacá: Duitama**. Road to páramo de la Rusia, 22 km from Duitama, before ‘fábrica de arepas Buenos Aires’, 5.92656N, 73.08826W, alt. 3650 m, 24 September 2013, *Morales-Briones D.F. & Uribe-Convers S. 506.* (holotype: ID!, isotypes: ANDES!, QCA!).

##### Description.

Ascendent subshrubs; stems erect to slightly decumbent, up to 22–27 cm long, robust, densely sericeous-villous, branched at apex. Basal stipules usually caducous, if present 5–6 mm long, adnate to the petiole at base, free at apex, entirely membranous, brown. Basal leaves usually caducous, if present 3–6-lobed, 4–6 × 3–6 mm; basal petiole 3–4 mm long. Distal leaves reduced, adnate, and connate to the distal stipules forming verticillate, lobed sheaths; sheath lobes 7–8, ascending to spreading at maturity, lanceolate, one lobe 3–5 lobate, lobes (4) 6–8 × 1–2 (4) mm, (2/3 of the entire sheath length), chartaceous, margin revolute, lower surface appressed sericeous-villous to villous, upper surface sericeous-villous to glabrescent. Inflorescences terminal or axilar ± glomerulate cymes; floral bracts lobed, ascending; 8–10 flowers per inflorescence; pedicels 0.3–0.4 mm long, pilose. Flowers 1.5–2.5 mm long; hypanthium turbinate-campanulate, brown-reddish at base, pilose-sericeous outside slightly glabrescent at base, glabrous within; 4 episepals and 4 sepals connivent to ± straight, abaxially sericeous, adaxially glabrous; episepals narrowly ovate, 0.8–1 × 0.2–0.3 mm, apex acute; sepals ovate, 0.5–1 × 0.4–0.5 mm, apex ± acute; stamens 2 adnate to the floral disc; carpels 2 (3), stigma clavate. Two achenes 0.8–0.9 × ca. 0.5 mm, subovoid.

**Figure 3.**
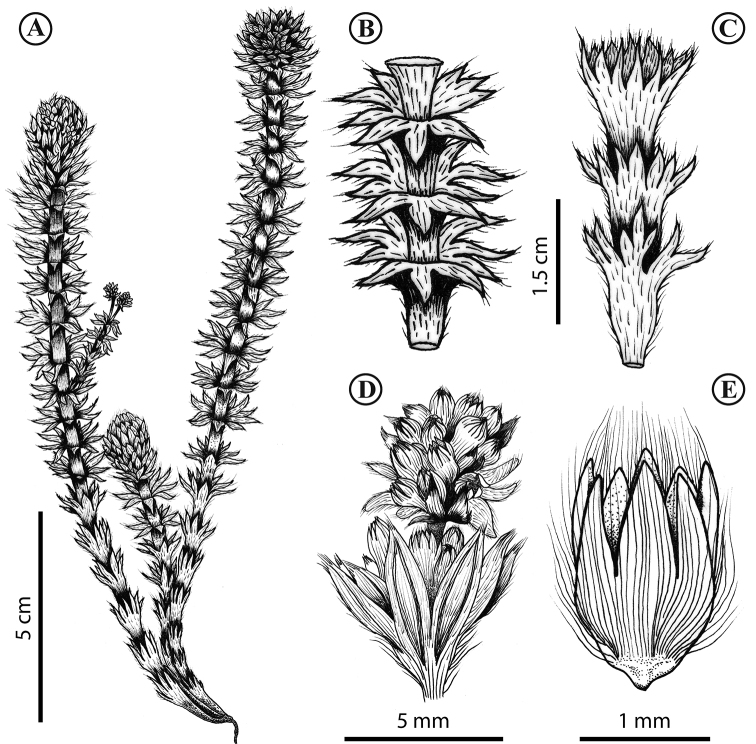
*Lachemillarothmaleriana*. **A** Habit **B** stem, apical leaves **C** stem, basal leaves **D** flowering branch **E** flower. Illustration by S. Cordero.

##### Distribution and ecology.

*Lachemillarothmaleriana* has a scattered distribution in the northcentral region of the Cordillera Oriental and in the southern part of the Cordillera Central of the Colombian Andes, between 3250 and 3768 m (Fig. 6). This species is mainly found in páramos dominated by bunchgrasses (*Agrostis*, *Calamagrostis*, *Chusquea*) and lives in sympatry with *L.hipidula*, *L.nivalis*, and *L.purdiei*. Flowering and fruiting collections dated from the months of January, May, September, and December.

##### Etymology.

The specific epithet honors Prof. Dr. Werner Rothmaler (1908–1962), a German botanist who studied *Lachemilla* in detail and described over 20 species of this genus.

##### Conservation status.

*Lachemillarothmaleriana* is known only from the three localities that are zones impacted by human activities, including conversion to agriculture. Following the IUCN (2017) guidelines, based on the reduced geographic distribution and altered land use at the type locality, this species should be categorized as vulnerable (VU).

**Figure 4.**
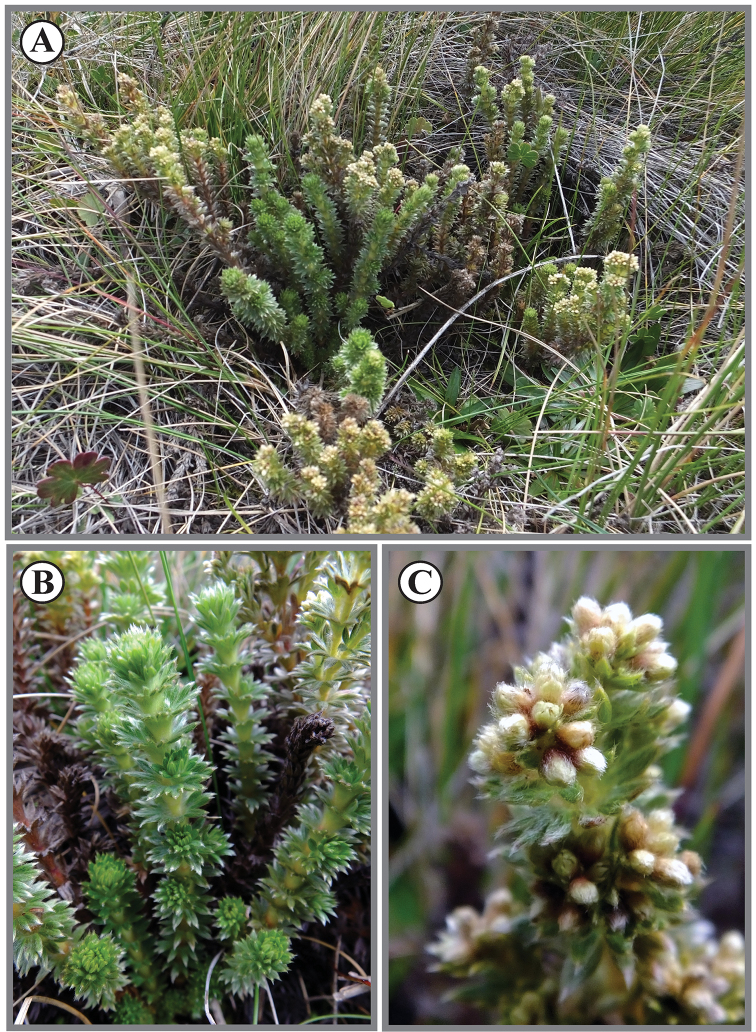
*Lachemillarothmaleriana*. **A** Habit **B** stems **C** flowering branch.

##### Additional specimens examined.

**COLOMBIA. Putumayo**: Comisaria del Putumayo, Alta cuenca del río Putumayo, filo de Cordillera entre El Encano y Sibundoy, páramo de San Antonio del Bordoncillo, 1.18333N, 77.1000W, alt. 3250 m, 4 January 1941, *Cuatrecasas J. 11761* (COL, F, JE frag.). **Santander**: Páramo del Consuelo, Belén, Vereda de San José, 18 km from Belén on road to Encino, 6.02920N, 72.96523W, alt. 3768 m, 23 September, 2013, *Morales-Briones D.F. & Uribe-Convers S. 492* (ANDES, ID, QCA).

##### Notes.

*Lachemillarothmaleriana* resembles *L.hispidula* and *L.nivalis* by its habit and erect stems with reduced leaves that fuse with the stipules to form verticillate sheaths, but differs by having trilobate lateral lobes. Additionally, *L.hispidula* has an overall hispid pubescence, while *L.rothmaleriana* has a characteristic sericeous-villous indumentum. Moreover, *L.rothmaleriana* has a turbinate-campanulate hypanthium with pilose-sericeous pubescence, while *L.hispidula* has a globose-campanulate hypanthium with pilose-hirsute pubescence. *Lachemillanivalis* has a hypanthium with similar indumentum but its shape is only slightly campanulate. In the shape of sheath lobes, *L.rothmaleriana* somewhat resembles *L.galioides* (Benth.) Rothm., but the latter has slender stems, broader sheath lobes, and villous-hispid indumentum.

**Figure 5.**
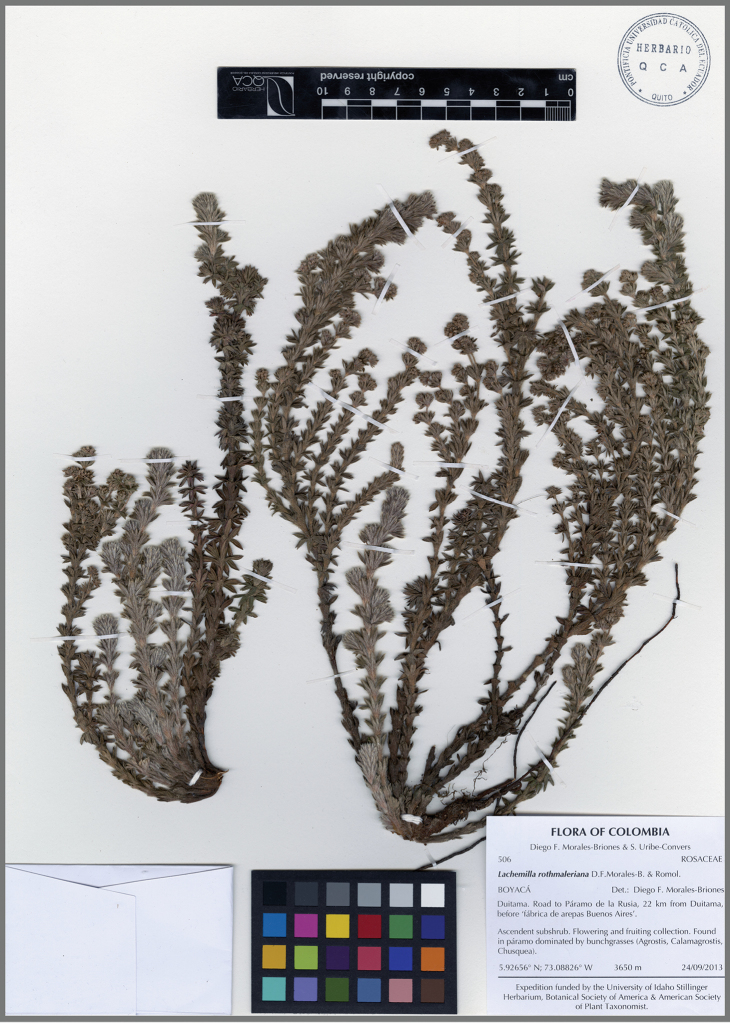
*Lachemillarothmaleriana*. Isotype collection: *Morales-Briones D.F. & Uribe-Convers S. 506.* (QCA).

**Figure 6.**
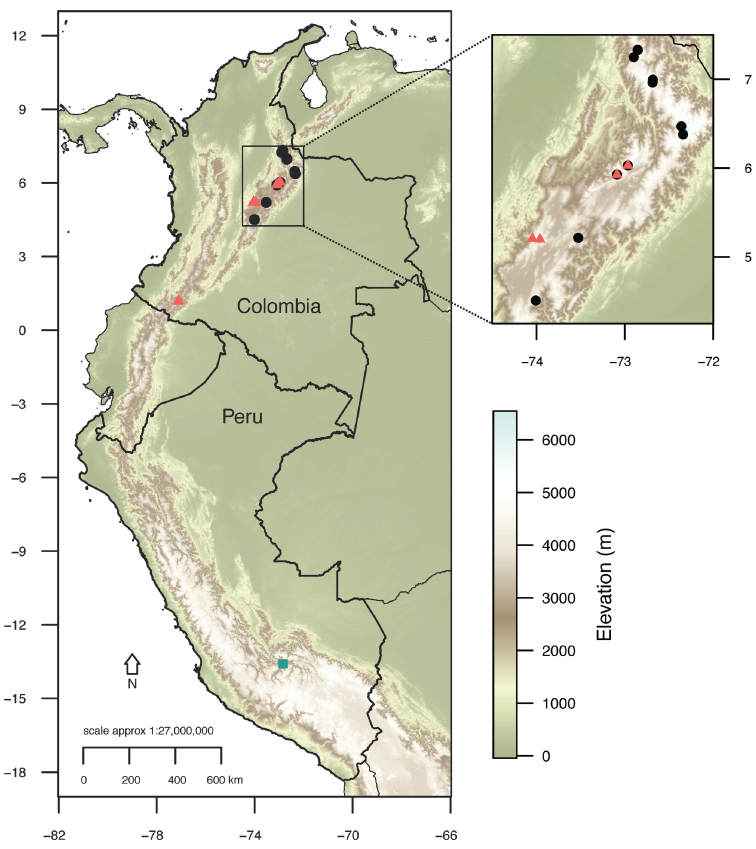
Geographic distribution of *Lachemillarothmaleriana* (read triangles), *L.argentea* (black circles), and *L.cyanea* (cyan rectangle).

#### Lachemilla argentea

Taxon classificationPlantaeRosalesLachemilla

D.F.Morales-B. & Romol. sp. nov.

urn:lsid:ipni.org:names:77199641-1

Figs 7 , 8 , 9

##### Diagnosis.

*Lachemillaargentea* differs from *L.holmgrenii* Rothm. and *L.adscendens* (Rothm.) Rothm. by its herbacecous habit with decumbent branches, conspicuous basal reniform tripartite leaves with an adaxial silvery villous pubescence, and a turbinate to urceolate hypanthium with pilose-sericeous pubescence.

##### Type.

**COLOMBIA. Bogotá, Distrito Capital**: Páramo de Cruz Verde. Path to Laguna El Verjón, 4.56102N, 74.02172W, alt. 3495 m, 26 September 2013, *Morales-Briones D.F. & Uribe-Convers S. 523* (holotype: ID!, isotypes: ANDES!, QCA!).

##### Description.

Decumbent herbs; branches decumbent up to 12 cm long, sericeous-villous. Basal stipules 5–10 mm long, adnate to the petiole, sparsely villous, membranous, brown at base, free at apex. Basal leaves tri-parted, blade reniform in outline, 6–7 (–10) × 7.5–10 (–1.3) mm, 3 lobes, chartaceous, slightly plicate, lobes unequally obovate-rhomboid, margin dentate-incised, teeth 3–6 on each of the lobes, lower surface sparsely sericeous-villous, upper surface villous; basal petiole 5–12 mm long. Distal stipules and distal leaves reduced adnate and connate, forming verticillate lobed sheaths; sheath lobes 6–10 ascending or slightly spreading; lobes 4–7 × 1.5–3 mm, lanceolate, entire or trilobed, decreasing in size. Inflorescences axilar or terminal glomerulate cymes, flowers aggregate at the distal part. Floral bracts 2–4 mm long, free, incised, and ascending; 3–7 flowers per inflorescence; pedicels 0.5–1 mm long, slightly pilose. Flowers 2–2.5 mm long; hypanthium turbinate to urceolate, green or reddish, pilose-sericeous outside, glabrous within; 4 episepals and 4 sepals green or slightly reddish, straight, abaxially pilose-sericeous, adaxially glabrous; episepals triangular, 0.7–0.8 × 0.4–0. 5 mm, apex acute; sepals triangular-ovate, 0.75–0.85 × 0.5–0.6 mm, apex acute; stamens 2 adnate to the floral disc; carpels 2–3, stigma clavate. Two achenes, ca. 1–1.5 × 0.6–0.8 mm, globose-ovoid.

**Figure 7.**
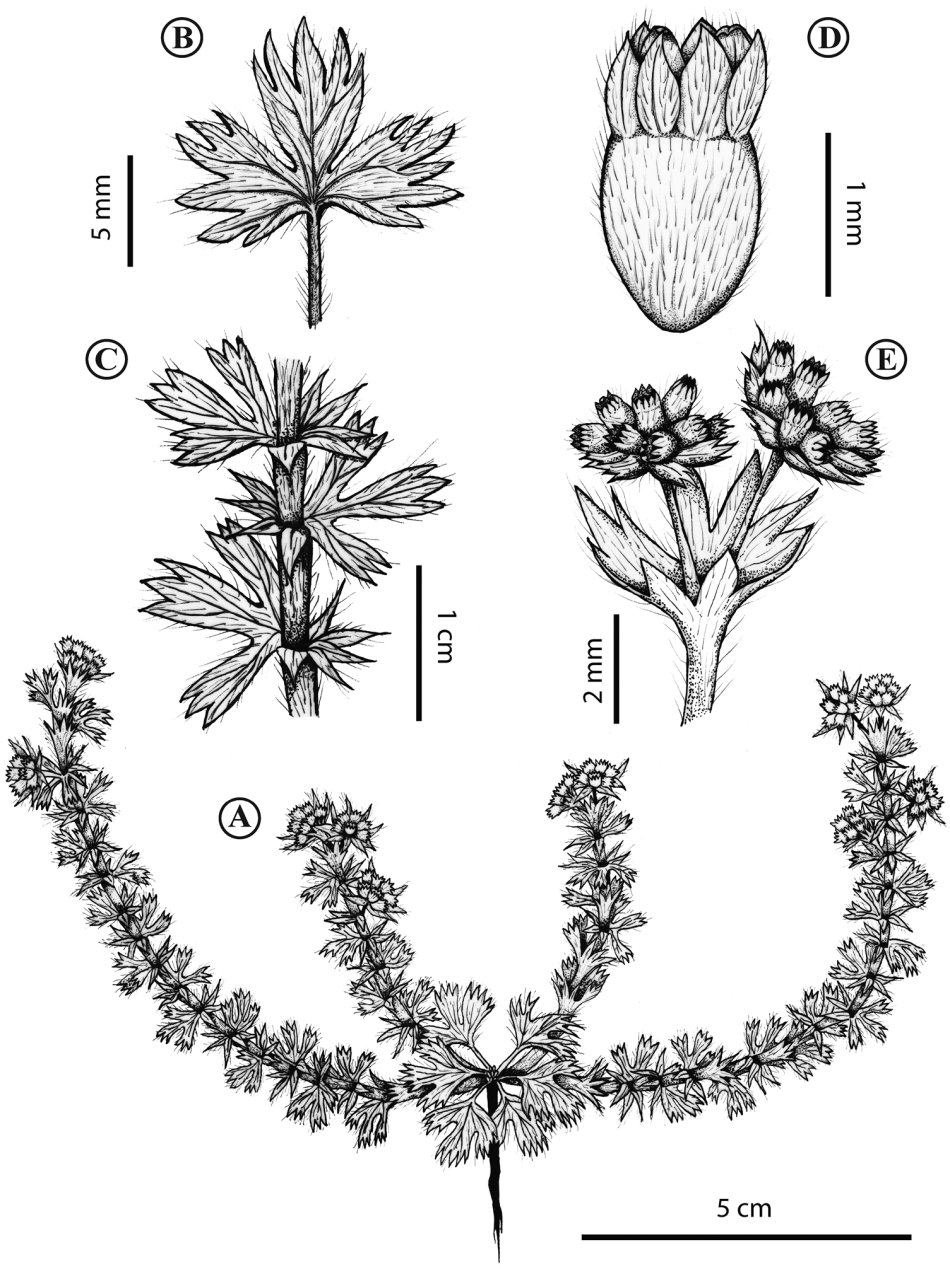
*Lachemillaargentea*. **A** Habit **B** basal leaf **C** stem, apical leaves **D** flower **E** flowering branch. Illustration by C. Rodríguez.

##### Additional specimens examined.

**COLOMBIA. Bogotá, Distrito Capital**: Páramo de Cruz Verde, Bogotá-Choachí road, km 11.2, alt. 3257 m, 1 May 1972, *Cleef A. 3330A* (COL). **Bogotá, Distrito Capital**: Páramo de Cruz Verde, path to Laguna El Verjón, 4.56102N, 74.02172W, alt. 3495 m, 26 September 2013, *Morales-Briones D.F. & Uribe-Convers S. 522* (ANDES, ID, QCA). **Boyacá**: Güicán, Sierra Nevada del Cocuy, road to the small house, in ‘Lagunilla’ area, towards to Púlpito del Diablo, 6.37906N, 72.33995W, alt. 3950 m, 18 September 2013, *Morales-Briones D.F. et al. 476* (ANDES, ID, QCA). **Boyacá**: Duitama, road to Páramo de la Rusia, 22 km from Duitama, before ‘fábrica de arepas Buenos Aires’, 5.92656N, 73.08826W, alt. 3650 m, 24 September 2013, *Morales-Briones D.F. & Uribe-Convers S. 507* (ANDES, ID, QCA). **Boyacá**: Belén, Vereda de San José, Páramo del Consuelo, 18 km from Belén, on the way to Encino, 6.02920N, 72.96523W, alt. 3768 m, 23 September 2013, *Morales-Briones D.F. & Uribe-Convers S. 499* (ANDES, ID, QCA). **Boyacá**: Páramo de Pisba, Socha-La Punta road, km 72, near to M.O.P campsite. El Cadillal, stony slope, alt. 3500 m, 8 June 1972, *Cleef A. 4235* (COL). **Boyacá**: Páramo NW of Belén, Vereda S. José de la Montaña, Alto de las Cruces and surroundings, alt. 3790 m, 24 February 1972, *Cleef A. 1756* (COL). **Boyacá**: Páramo de Pisva, Socha-La Punta road, km 61, 5.6 km east of Los Pinos, Alto de Granados, alt. 3635 m, 12 June 1972, *Cleef A. 445813* (COL). **Boyacá**: Páramo de Pisva, flank SW of Morros de S. Gabriel, 2 km SW of Laguna Batanera, alt. 3670 m, 18 June 1972, *Cleef A. 4702A* (COL). **Cundinamarca**: Villa Pinzón, Páramo de Guachenque. Entrance to the Laguna del Valle and surroundings of Laguna del Mapa, 5.21641N, 73.52675W, alt. 3346 m, 25 September 2013, *Morales-Briones D.F. & Uribe-Convers S. 514* (ANDES, ID, QCA). **Cundinamarca**: Páramo de Palacio, 18 km from the road, alt. 3485 m, 16 December 1971, *Cleef A. 327* (COL) . **Santander**: Vetas, road to the Laguna Pajaritos, at the entrance of private property, 7.33349N, 72.85373W, alt. 3539 m, 14 September 2013, *Morales-Briones D.F. et al. 437* (ANDES, ID, QCA). **Santander**: Vetas, road to Laguna Pajaritos, 7.33086N, 72.85106W, alt. 3585 m, 14 September 2013 *Morales-Briones et al. D.F. 440* (ANDES, ID, QCA). **Santander**: Páramo de Almorzadero, road Presidente-Cerrito, km 98, 6.99470N, 72.68187W, alt. 3567 m, 16 September 2013, *Morales-Briones D.F. et al. 458* (ANDES, ID, QCA) . **Santander**: Páramo de Almorzadero, road Presidente-Cerrito, km 98, 6.96333N, 72.68488W, alt. 3801 m, 16 September 2013, *Morales-Briones D.F. & Uribe-Convers S. 459* (ANDES, ID, QCA).

**Figure 8.**
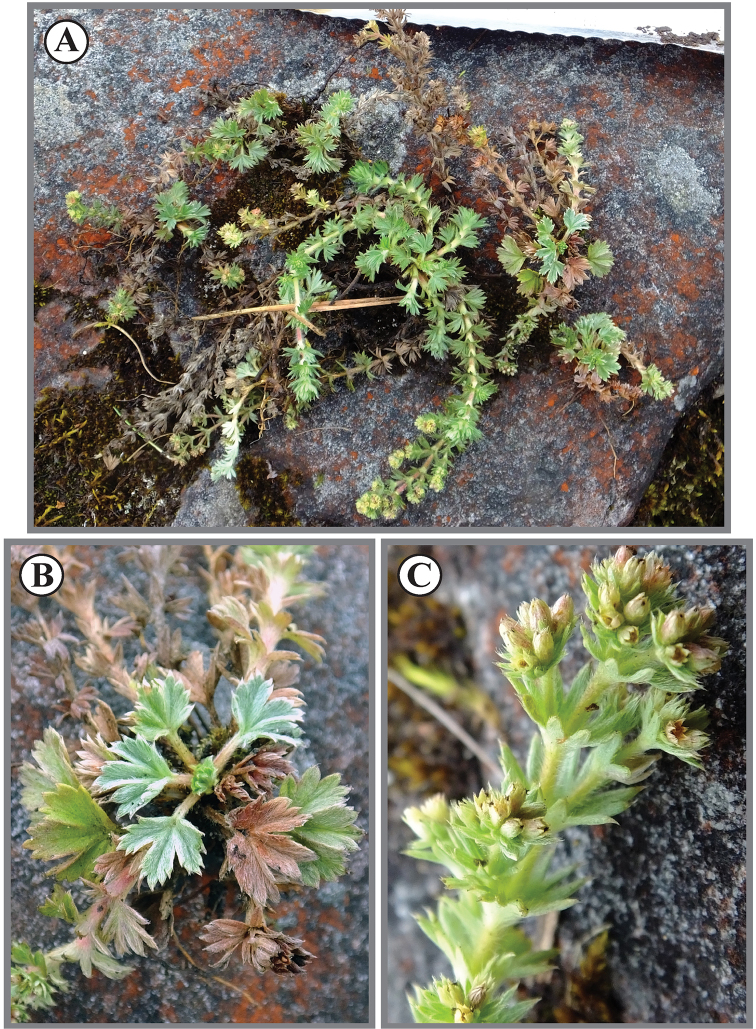
*Lachemillaargentea*. **A** Habit **B** basal leaves **C** flowering branch.

##### Mixed specimens examined.

the following specimens are collections with two different species under the same number, one (a) correspond to *L.argentea*. **Boyacá**: Páramo de Pisva, Alto de Granados, 5 km E of Los Pinos, alt. 3735m, 15 June 1972, *Cleef A. 4593A* (a) (COL). **Boyacá**: Güicán Sierra Nevada del Cocuy, after Cabañas Kanwara, 17.7 km from ‘water plant’, Lomas Las Pajas, 6.47093N, 72.35934W, alt. 4105 m, 17 September 2013, *Morales-Briones D.F. & Uribe-Convers S. 473* (a) (ANDES, ID, QCA). **Santander**: Páramo de Santurbán, Berlín, 7 km from Vetas, 7.24935N, 72.89784W, alt. 3567 m, 13 September 2013, *Morales-Briones D.F. et al. 431* (a) (ANDES, ID, QCA).

##### Deviating specimens examined.

The following specimens have similar habit, leaf shape, and pubescence to *L.argentea*, but they only differ in having glabrous flowers. These specimens may represent only a variety of *L.argentea*, but they are maintained here as uncertain taxa until more material is available or can be included in phylogenetic analyses. **Boyacá**: Páramo NW of Belén, Quebrada Minas. Hoya CLLA, Slopes N of Valle Lajas, alt. 3835m, 2 March 1973, *Cleef A. 2119A* (COL). **Boyacá**: Páramo de Pisva, Road Socha-La Punta, km 61.5, 6 km E from Los Pinos Alto de Granados, alt. 3630 m, 14 June 1972, *Cleef A. 4545* (COL). **Cundinamarca**: Páramo de Palacio aprox. 1 km E from ‘la mina de cal’, alt. 3853 m, 19 May 1972, *Cleef A. 3853A* (COL). **Quidio/Tolima**: Paramillo of Quindio and Páramo de Tolima. 13 km from Valle de Cocora. 4.64433N, 75.43060W, alt. 3645 m, 4 October 2013, *Morales-Briones D.F. et al. 543* (ANDES, ID, QCA). **Santander**: Vetas, road to Laguna Pajaritos, 7.33086N, 72.85106W, alt. 3585 m, 14 September 2013, *Morales-Briones D.F. et al. 439* (ANDES, ID, QCA).

##### Distribution and ecology.

*Lachemillaargentea* is distributed in the central northern (primarily) regions of the Cordillera Oriental between 3275 and 3735 m (Fig. 6). This species occurs in humid and very humid páramos dominated by grasses, shrubs, and dwarf shrubs. *Lachemillaargentea* can be found living in sympatry with multiple species of *Lachemilla*, including *L.aphanoides*, *L.hispidula*, *L.nivalis*, *L.mandoniana* (Wedd.) Rothm., *L.purdiei*, and *L.vulcanica* (Schltdl. & Cham.) Rydb. Flowering and fruiting collections dated from the months of February, May, June, and September.

**Figure 9.**
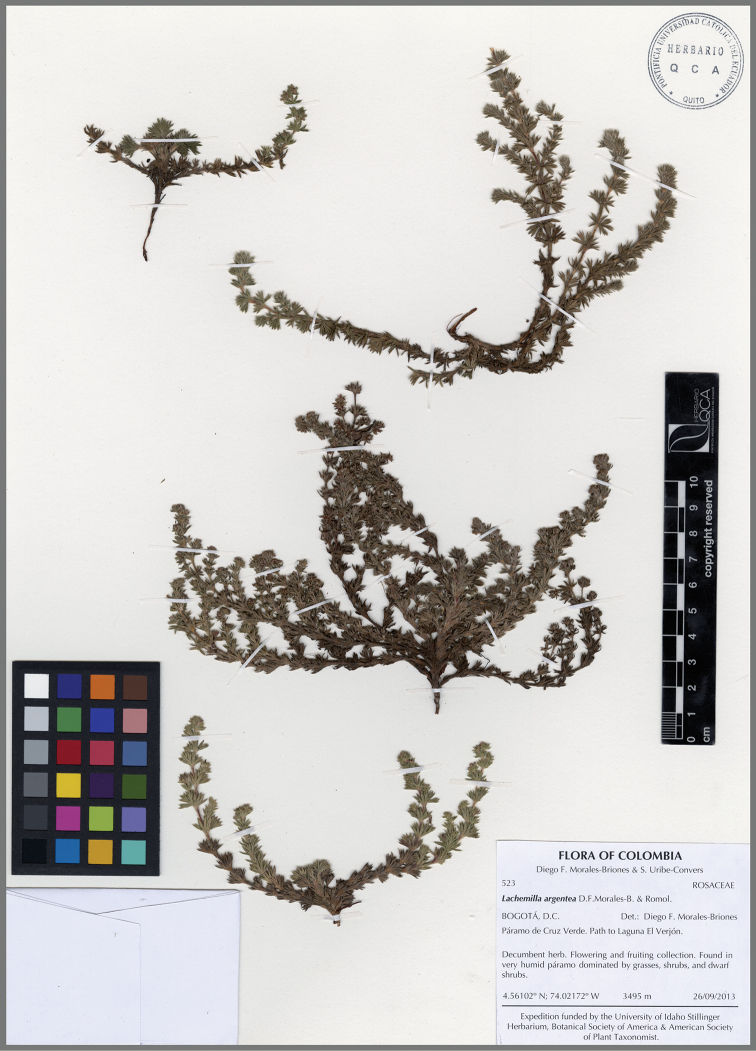
*Lachemillaargentea*. Isotype collection: *Morales-Briones D.F. & Uribe-Convers S. 523* (QCA).

##### Etymology.

The specific epithet refers to the silvery aspect of the basal leaves.

##### Conservation status.

*Lachemillaargentea* is a common element throughout its distributional range, and occurs in several well-conserved areas. Following the IUCN (2017) guidelines, we consider this species is not at risk and should be categorized as least concern (LC). Nonetheless, the rapid deterioration and conversion to agriculture of proximate areas where *L.argentea* occurs may put this species at some threat in the near future.

##### Notes.

*Lachemillaargentea* resembles *L.holmgrenii* and *L.adscendens* by having basal tripartite leaves and stem leaves fused to the stipules forming verticillate sheaths with one tripartite lobe that is larger than the remaining lobes, but differs in having an herbaceous habit with decumbent branches, while the other two species are subshrubs with suberect to ascending branches. Furthermore, *L.argentea* has conspicuous basal reniform tripartite leaves with an adaxial silvery villous pubescence, while *L.holmgrenii* and *L.adscendens* have smaller hirsute leaves. These species also vary in the number of sheath lobes; *L.argentea* has 6–10 ascending or slightly spreading lobes, while *L.holmgrenii* and *L.adscendens* have 5–7 loosely ascending to reflexed lobes, and eight erecto-patent lobes, respectively. By having conspicuous basal leaves, *L.argentea* also resembles *L.sprucei*, but the latter is a subshrub with suberect stems and coriaceous 5-parted basal leaves, in addition to distinct 3-parted distal leaves.

### Key to *Lachemillarothmaleriana*, *L.argentea*, and similar species of the Verticillate clade from Colombia

**Notes.** Includes species with stem leaves fused to the stipules forming verticillate sheaths (sect. Nivales and sect. Subnivales; *sensu*Rothmaler 1935a). Accepted taxa and synonymy follows Morales-Briones et al. (2018a). Adapted in part from Perry (1929) and Romoleroux (2004).

**Table:** 

1	Subshrubs with erect or decumbent stems; basal leaves caducous or sessile when present; stem leaves reduced, with the adnate stipule forming verticillate sheaths along the entire stem	**2**
–	Herbs with procumbent or decumbent stems, or subshrubs with suberect or ± decumbent stems; basal leaves petiolate; lower stem leaves subequal to or often longer than the stipules, 3-parted or 3-lobed, uppermost stem leaves reduced, with the adnate stipule forming verticillate sheaths	**8**
2	Outer surface of sheath lobes glabrous or puberulent; lobes 4–6	**3**
–	Outer surface of sheath lobes conspicuously pubescent; lobes 4–15	**4**
3	Outer surface of sheath lobes glabrous and reticulate, margins strongly revolute	*** L. equisetiformis ***
–	Outer surface of sheath lobes hispidulous and not reticulate, margins revolute at apex	*** L. ericoides ***
4	Plants pilose, villous or sericeous; sheath lobes laxly ascending to erect, basal leaves mainly caducous	**5**
–	Plants hispid, hirsute or villous; sheath lobes widely spreading to abruptly reflexed; basal leaves usually present (except in *L.verticillata*)	**6**
5	Stems sparsely villous, pilose to glabrescent, or sericeous; sheath lobes 10–15, linear to linear-lanceolate, all lobes entire	*** L. nivalis ***
–	Stems densely sericeous-villous; sheath lobes 7–8, lanceolate, one lobe 3–5 lobate	*** L. rothmaleriana ***
6	Sheath lobes 7–8 (10); stems villous; sepals and episepals divergent and equal in length; hypanthium villous	*** L. verticillata ***
–	Sheath lobes 9–13; stems hispid or hirsute; sepals and episepals connivent, episepals usually a little shorter than the sepals	**7**
7	Plant copiously hirsute; sheath lobes reflexed; hypanthium glabrous	*** L. galioides ***
–	Plant sparsely hispid; sheath lobes spreading; hypanthium glabrous or pubescent	*** L. hispidula ***
8	Caespitose herbs; stems procumbent or slightly decumbent, multiple branched and rooting	**9**
–	Herbs or subshrubs; stems suberect or ± decumbent, not branched and not rooting	**10**
9	Flowers ca. 2–2.5 mm long, hypanthium campanulate, appressed villous, carpels 5–10; stigma clavate	*** L. holosericea ***
–	Flowers ca. 3.5 mm long, hypanthium globose-urceolate, sericeous-villous, carpels 5; stigma subclavate	*** L. purdiei ***
10	Basal leaves 5-parted, coriaceous; flowers ca. 2.5–3.5 long; carpels 5–6	*** L. sprucei ***
–	Basal leaves 3-parted, not coriaceous; flowers ca. 2–2.5 long; carpels 2–4	**11**
11	Herbs, sericeous-villous; stems decumbent; sheath lobes 6–10, ascending or slightly spreading	*** L. argentea ***
–	Subshrubs, villous or hirsute; stems suberect or ascending, sheath lobes 5–8, loosely ascending to reflex	**12**
12	Stems suberect; sheath lobes 5–7; hypanthium campanulate-globose; carpels 2–3	*** L. holmgrenii ***
–	Stems ascending; sheath lobes 8; hypanthium globose-urceolate; carpels 3–4	*** L. adscendens ***

#### Lachemilla cyanea

Taxon classificationPlantaeRosalesLachemilla

D.F.Morales-B. & Romol. sp. nov.

urn:lsid:ipni.org:names:77199642-1

Figs 10 , 11 , 12

##### Diagnosis.

*Lachemillacyanea* differs from *L.ranunculoides* (L.M. Perry) Rothm. and *L.williamsii* (L.M. Perry) Rothm by its hirsute pubescence, reniform basal leaves that have a blue-green color, and turbinate-campanulate hypanthium.

##### Type.

**PERU. Apurímac**: Abancay Province, road Abancay - Cuzco, 23 km from Abancay, 13.59722S, 72.84083W, alt. 3423 m, 25 June 2012, *Morales-Briones D.F. & Uribe-Convers S. 246*, (holotype: ID!, isotypes: HAO!, QCA!).

##### Description.

Rosette herbs up to 10 cm long, branches decumbent, sparsely hirsute. Basal stipules 10 mm long, adnate to the petiole, sparsely hirsute, membranaceous, brown at base, free at apex. Basal leaves tri-parted, blade reniform in outline, 17–20 × 25–26 mm, 3 lobes, chartaceous, slightly plicate, lobes unequally obovate-rhomboid, lateral lobes divided the length 1/2 of the blade, margin dentate-incised, teeth 8–10 on each of the lobes, lower and upper surface sparsely hirsute-villous; basal petiole 12–20 mm long. Distal stipules 7–8 mm long, connate and adnate to the petiole at base, free and incised at apex, leaf-like in texture. Distal leaves tri-parted, 8–10 × 8–12 mm, lobes obovate-rhomboid, decreasing in size; distal petiole 1–2.5 mm long. Inflorescences axilar or terminal glomerulate cymes, flowers aggregate at the distal part. Floral bracts 1–2 mm long, free, incised, and ascending to slightly spreading; 2–6 flowers per inflorescence; pedicels 0.6–1 mm long, sericeous. Flowers 2–2.5 mm long; hypanthium turbinate-campanulate, green, glabrous outside and within; 4 episepals and sepals green to reddish at apex, straight, abaxially and adaxially glabrous; episepals triangular, 0.7–0.8 × 0.3–0.4 mm, apex acute; sepals triangular-ovate, 0.7–0.8 × 0.5 mm, apex acute; stamens 2 adnate to the floral disc; carpels 2, stigma clavate. Two achenes, ca. 1 × 0.7–0.8 mm, globose-ovoid.

**Figure 10.**
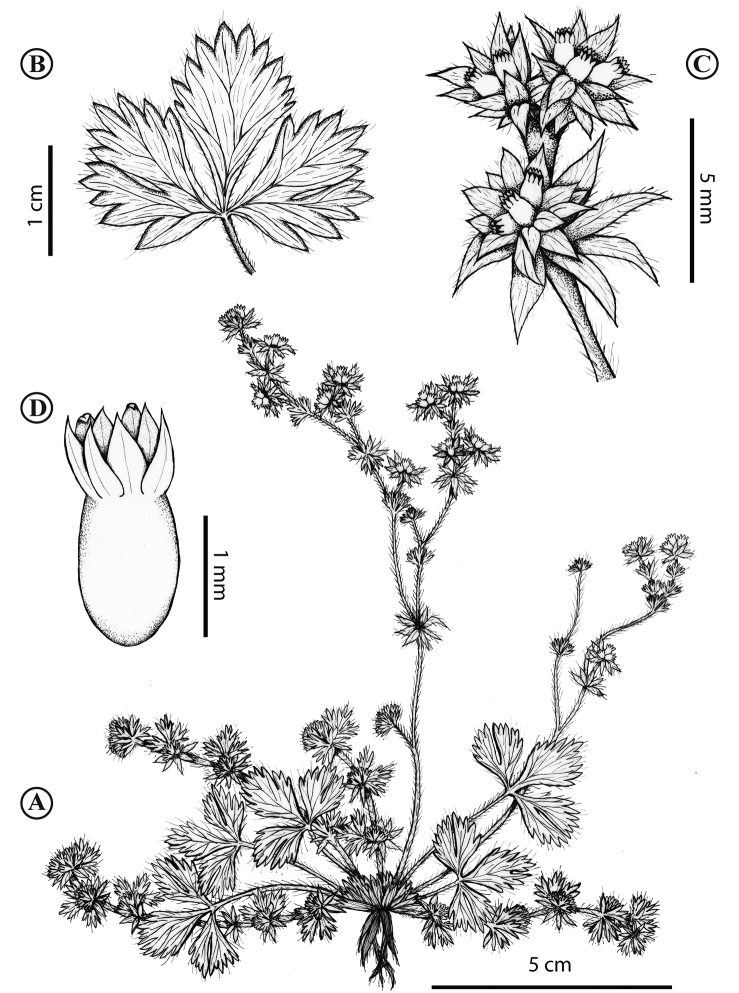
*Lachemillacyanea*. **A** Habit **B** basal leaf **C** flowering branch **D** flower. Illustration by C. Rodríguez.

##### Distribution and ecology.

*Lachemillacyanea* is only known from the Province of Abancay in the Department of Apurímac at ca. 3420 m in southern Peru (Fig. 6). This species occurs in the transition zone between the montane forest and the high-elevation grassland dominated by dwarf shrubs and herbs. This species lives in sympatry with *L.aphanoides* and *L.fulvescens*. It was collected in flower and fruit in late June.

##### Etymology.

The specific epithet refers to the blue-green color of the leaves.

##### Conservation status.

*Lachemillacyanea* is only known from the type locality in a zone severely impacted by human activities, including conversion to agriculture. Following the IUCN (2017) guidelines, based on the reduced geographic distribution and altered land use at the type locality, this species should be categorized as endangered (EN), at least until other populations are discovered.

**Figure 11.**
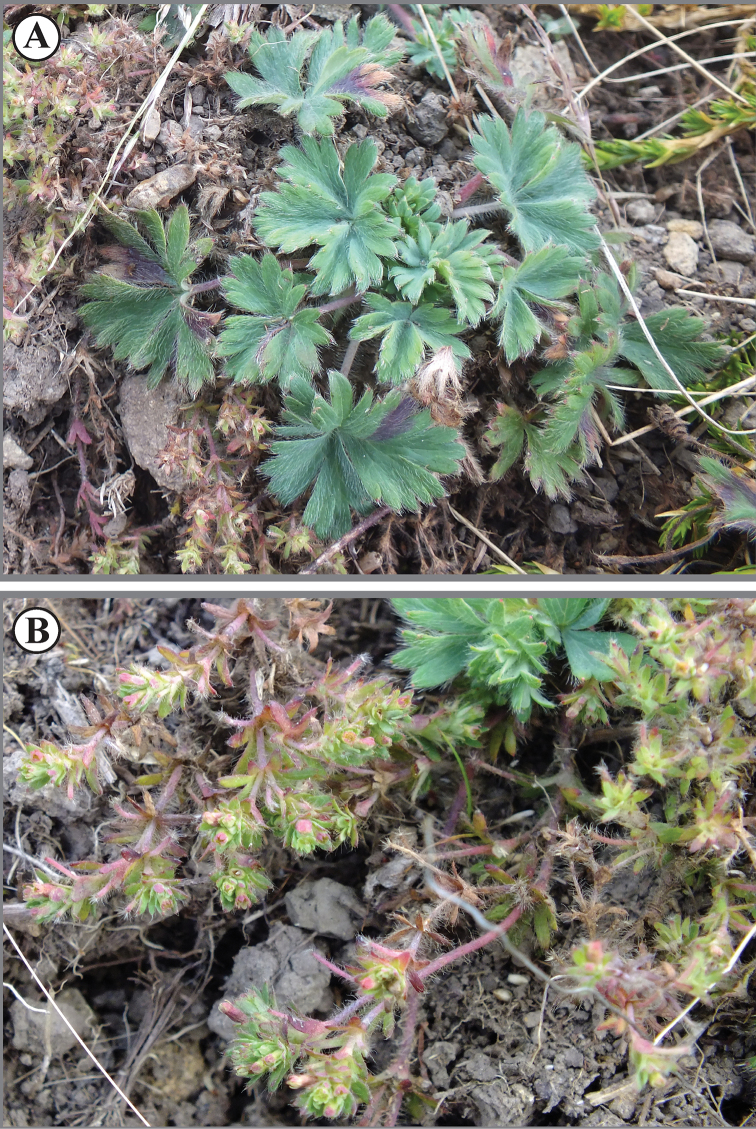
*Lachemillacyanea*. **A** Habit **B** flowering branch.

##### Notes.

*Lachemillacyanea* resembles *L.ranunculoides* in habit and glabrous flower, but differs in the reniform shape of the basal leaves in contrast to the orbicular-ovate shape of the latter. Moreover, *L.cyanea* has a turbinate-campanulate hypanthium while *L.ranunculoides* has an oblong-ventricose hypanthium. *Lachemillacyanea* also resembles *L.aphanoides* in the tripartite basal leaves and glomerulate inflorescence, but differs in the rosette habit and decumbent branches, in contrast to the erect stems of *L aphanoides. Lachemillacyanea* is also similar to *L.williamsii* in habit, but the former has an overall hirsute pubescence and glabrous flowers with strictly two stamens, while the latter has a general villous pubescence, and villous flowers with up to four stamens (a characteristic only known in *L.williamsii*).

**Figure 12.**
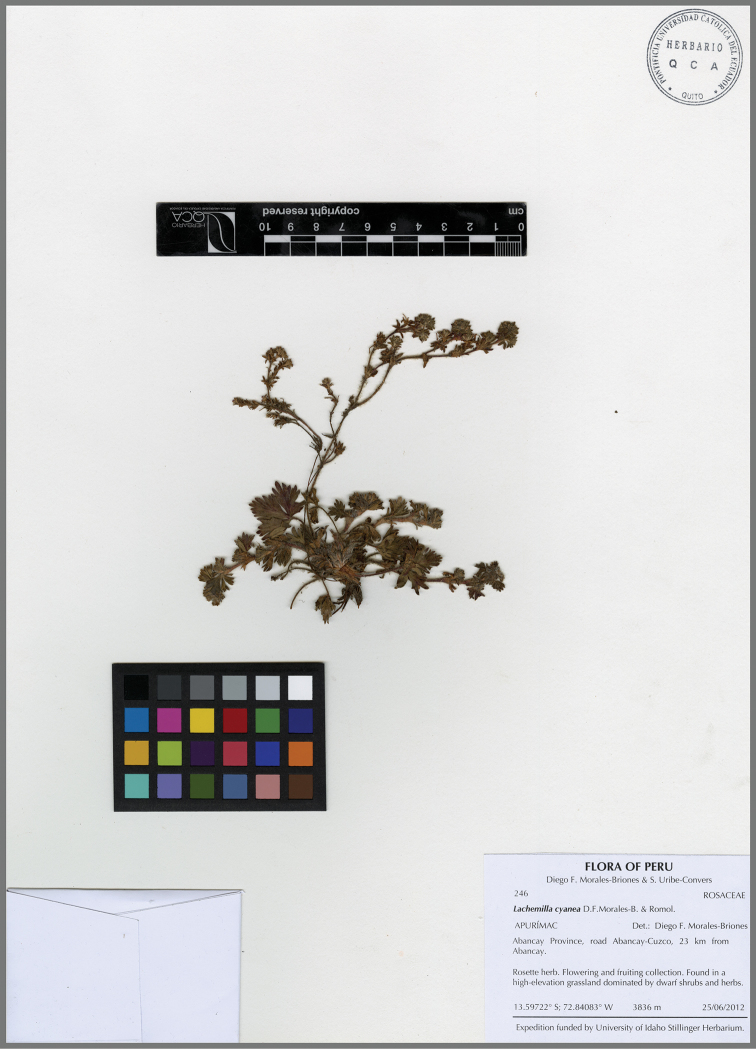
*Lachemillacyanea*. Isotype collection: *Morales-Briones D.F. & Uribe-Convers S. 246* (QCA).

### Key to *Lachemillacyanea* and similar species from the Central Andes

**Notes.** Includes species with a general prostrate habit, tripartite basal leaves, and mainly glomerulate inflorescences (series *Aphanoides*, subseries 2; *sensu*Perry 1929). Accepted taxa and synonymy follows Morales-Briones et al. (2018a). Adapted from Perry (1929).

**Table:** 

1	Herbs with creeping stems, mat-forming; lateral segments of the leaves entire; inflorescences loosely dichotomous; stipules entirely membranaceous	*** L. rupestris ***
–	Herbs with prostrate or loosely decumbent stems, villous or hirsute; lateral segments of the leaves variously lobed; inflorescences glomerulate cymes; stipules herbaceous at least at apex	**2**
2	Basal leaves lobes acute; flowers ca. 3 mm long, hirsute-villous to sericeous; stamens 2–4	*** L. williamsii ***
–	Basal leaves lobes obtuse; flowers ca. 2–2.5 mm long, glabrous or with scattering hairs on the hypanthium lobes; stamens 2	**3**
3	Plants densely sericeous-villous; sepals oblong-ovate	*** L. grisebachiana ***
–	Plants hirsute or glabrate; sepals ovate	**4**
4	Basal leaves subreniform; cauline leaves mainly subsessile; episepals reduced or absent; carpels 1–2	*** L. frigida ***
–	Basal leaves orbicular or reniform; cauline leaves mainly petiolate; episepals conspicuous, carpels 2–4	**5**
5	Basal leaf blade reniform; hypanthium turbinate-campanulate; carpels 2	*** L. cyanea ***
–	Basal leaf blade orbicular; hypanthium oblong-ventricose; carpels 2–4	*** L. ranunculoides ***


## Supplementary Material

XML Treatment for Lachemilla rothmaleriana

XML Treatment for Lachemilla argentea

XML Treatment for Lachemilla cyanea

## Appendix I

**Species and vouchers used in the phylogenetic analyses. Arranged by *species name* and authority, DNA number, and *collector and number* (herbarium code).**

**Outgroup: *Alchemillajohnstonii*** Oliv.: 2015_358, *B. Gehrke 364* (UZH). ***Alchemillamollis*** (Buser) Rothm.: 2012_228_US, *D.F. Morales-Briones 687* (ID). ***Aphanescotopaxiensis*** Romol. & Frost-Ols.: 2013_004_EC, *D.F. Morales-Briones 276* (ID). ***Farinopsissalesoviana*** (Stephan) Chrtek & Soják: 2016_580, *B. Bartholomew 8364* (MO). **Ingroup: *Lachemillaadscendens*** (Rothm.) Rothm.: 2013_445_CO, *D.F. Morales-Briones & S. Uribe-Convers 429A* (ID). ***Lachemillaandina*** (L.M. Perry) Rothm.: 2012_249_PE, *D.F. Morales-Briones & S. Uribe-Convers 218* (ID); 2012_379_EC, *D.F. Morales-Briones et al. 122* (QCA). ***Lachemillaaphanoides*** (Mutis ex L. f.) Rothm.: 2012_246_PE, *D.F. Morales-Briones & S. Uribe-Convers 215* (ID); 2012_396_EC, *D.F. Morales-Briones et al. 115* (QCA); 2013_446_CO, *D.F. Morales-Briones & S. Uribe-Convers 430* (ID); 2014_541_CO, *D.F. Morales-Briones & S. Uribe-Convers 550* (ID). ***Lachemillaargentea*** D.F. Morales-B. & Romol.: 2013_447_CO, *D.F. Morales-Briones et al. 431* (ID); 2013_475_CO, *D.F. Morales-Briones et al. 459* (ID); 2014_507_CO, *D.F. Morales-Briones & S. Uribe-Convers 514* (ID); 2014_516_CO, *D.F. Morales-Briones & S. Uribe-Convers 523* (ID). ***Lachemillabarbata*** (C. Presl) Rothm.: 2012_260_PE, *D.F. Morales-Briones & S. Uribe-Convers 229* (ID). ***Lachemillabipinnatifida*** (L.M. Perry) Rothm.: 2013_428_BO, *D.F. Morales-Briones et al. 326* (ID). ***Lachemillacyanea*** D.F. Morales-B. & Romol.: 2012_277_PE, *D.F. Morales-Briones & S. Uribe-Convers 246* (ID). ***Lachemilladiplophylla*** (Diels) Rothm.: 2012_229_PE, *D.F. Morales-Briones & S. Uribe-Convers 198* (ID). ***Lachemillaequisetiformis*** (Trevir.) Rothm.: 2012_108_VE, *A.J.P. Martin 740* (QCA). ***Lachemillaerodiifolia*** (Wedd.) Rothm.: 2012_244_PE, *D.F. Morales-Briones & S. Uribe-Convers 213* (ID). ***Lachemillafrigida*** (Wedd.) Rothm.: 2012_300_PE, *D.F. Morales-Briones & S. Uribe-Convers 269* (ID). ***Lachemillafulvescens*** (L.M. Perry) Rothm.: 2014_551_CO, *D.F. Morales-Briones & S. Uribe-Convers 560* (ID). ***Lachemillagalioides*** (Benth.) Rothm.: 2012_348_EC, *K. Romoleroux et al. 4403* (QCA). ***Lachemillahirta*** (L.M. Perry) Rothm.: 2012_317_EC, *K. Romoleroux et al. 4588* (QCA); 2012_343_EC, *K. Romoleroux et al. 4389* (QCA). ***Lachemillahispidula*** (L.M. Perry) Rothm.: 2012_310_EC, *K. Romoleroux et al. 4470* (QCA). ***Lachemillaholmgrenii*** Rothm.: 2012_347_EC, *K. Romoleroux et al. 4397* (QCA). ***Lachemillaholosericea*** (L.M. Perry) Rothm.: 2012_395_EC, *D.F. Morales-Briones & K. Romoleroux 161* (QCA); 2014_479_CO, *D.F. Morales-Briones et al. 485* (ID). ***Lachemillajamesonii*** (L.M. Perry) Rothm.: 2012_350_EC, *K. Romoleroux et al. 4387* (QCA). ***Lachemillajaramilloi*** Romol. & D.F. Morales-B.: 2012_006_EC, *D.F. Morales-Briones & S. Uribe-Convers 193* (ID); 2012_371_EC, *D.F. Morales-Briones et al. 115* (QCA). ***Lachemillalechleriana*** (Griseb.) Rothm.: 2012_271_PE, *D.F. Morales-Briones & S. Uribe-Convers 240* (ID). ***Lachemillallanganatensis*** Romol.: 2012_363_EC, *D.F. Morales-Briones et al. 105* (QCA). ***Lachemillamandoniana*** (Wedd.) Rothm.: 2012_370_EC, *D.F. Morales-Briones et al. 114* (QCA). ***Lachemillamexiquense*** D.F. Morales-B.: 2015_233_MX, *D.F. Morales-Briones & P. Tenorio-Lezama 683* (ID). ***Lachemillamoritziana*** Dammer: 2013_443_CO, *D.F. Morales-Briones & S. Uribe-Convers 427* (ID). ***Lachemillanivalis*** (Kunth) Rothm.: 2012_349_EC, *D.F. Morales-Briones & M.F. Latorre-Barragán 11* (QCA). ***Lachemillaorbiculata*** (Ruiz & Pav.) Rydb.: 2012_377_EC, *D.F. Morales-Briones et al. 121* (QCA). ***Lachemillapectinata*** (Kunth) Rothm.: 2012_326_EC, *K. Romoleroux et al. 4706* (QCA). ***Lachemillaperryana*** (Rothm.) Rothm.: 2012_380_EC, *D.F. Morales-Briones et al. 123* (QCA). ***Lachemillapinnata*** (Ruiz & Pav.) Rothm.: 2012_251_PE, *D.F. Morales-Briones & S. Uribe-Convers 220* (ID). ***Lachemillapolylepis*** (Wedd.) Rothm.: 2012_359_CO, *P. Sklenář FAA652* (PRC). ***Lachemillapringlei*** Rydb.: 2015_150_MX, *D.F. Morales-Briones & P. Tenorio-Lezama 595* (ID). ***Lachemillaprocumbens*** (Rose) Rydb.: 2015_200_MX, *D.F. Morales-Briones & P. Tenorio-Lezama 648* (ID). ***Lachemillapseudovenusta*** (Rothm.) Rothm.: 2012_275_PE, *D.F. Morales-Briones & S. Uribe-Convers 244* (ID). ***Lachemillapurdiei*** (L.M. Perry) Rothm.: 2014_501_CO, *D.F. Morales-Briones & S. Uribe-Convers 508* (ID). ***Lachemillaranunculoides*** (L.M. Perry) Rothm.: 2012_240_PE, *D.F. Morales-Briones & S. Uribe-Convers 209* (ID). ***Lachemillarepens*** (C. Presl) Rothm.: 2012_263_PE, *D.F. Morales-Briones & S. Uribe-Convers 232* (ID). ***Lachemillarothmaleriana*** D.F. Morales-B. & Romol.: 2014_486_CO, *D.F. Morales-Briones & S. Uribe-Convers 492* (ID); 2014_499_CO, *D.F. Morales-Briones & S. Uribe-Convers 506* (ID). ***Lachemillarupestris*** (Kunth) Rothm.: 2012_394_EC, *D.F. Morales-Briones et al. 157* (QCA). ***Lachemillasarmentosa*** (L.M. Perry) Rothm.: 2013_396_BO, *D.F. Morales-Briones et al. 292* (ID); 2013_413_BO, *D.F. Morales-Briones et al. 309* (ID). ***Lachemillasibbaldiifolia*** (Kunth) Rydb.: 2015_154_MX, *D.F. Morales-Briones & P. Tenorio-Lezama 599* (ID). ***Lachemillasprucei*** (L.M. Perry) Rothm.: 2012_340_EC, *K. Romoleroux et al. 4474* (QCA). ***Lachemillastandleyi*** (L.M. Perry) Rothm.: 2016_614_CR, *K. Romoleroux et al. 5023* (QCA). ***Lachemillasteinbachii*** Rothm.: 2013_415_BO, *D.F. Morales-Briones et al. 311* (ID). ***Lachemillatalamanquensis*** Romol. & D.F. Morales-B.: 2012_334_CR, *K. Romoleroux et al. 5008* (QCA). ***Lachemillatanacetifolia*** Rothm.: 2012_331_EC, *K. Romoleroux et al. 4396* (QCA). ***Lachemillauniflora*** Maguire: 2012_362_EC, *D.F. Morales-Briones et al. 75* (QCA); 2012_385_EC, *D.F. Morales-Briones et al. 130* (QCA). ***Lachemillavelutina*** (S. Watson) Rydb.: 2015_162_MX, *D.F. Morales-Briones & P. Tenorio-Lezama 607* (ID). ***Lachemillavenusta*** (Schltdl. & Cham.) Rydb.: 2015_157_MX, *D.F. Morales-Briones & P. Tenorio-Lezama 602* (ID). ***Lachemillaverticillata*** (Fielding & Gardner) Rothm.: 2012_339_CR, *K. Romoleroux et al. 5007* (QCA). ***Lachemillavulcanica*** (Schltdl. & Cham.) Rydb.: 2012_250_PE, *D.F. Morales-Briones & S. Uribe-Convers 219* (ID); 2015_171_MX, *D.F. Morales-Briones & P. Tenorio-Lezama 617* (ID). ***Lachemillawilliamsii*** (L.M. Perry) Rothm.: 2013_402_BO, *D.F. Morales-Briones et al. 298* (ID).
